# First Report on Atrial Leadless Pacing in a Lateral Tunnel Fontan Patient

**DOI:** 10.1111/jce.70215

**Published:** 2026-01-16

**Authors:** Daniel Cortez, Omar Abu Anza, Mohammad Alnoor

**Affiliations:** ^1^ Department of Pediatric Cardiology University of California at Davis Sacramento California USA

**Keywords:** atrial pacing, Fontan, leadless pacemaker

## Abstract

**Introduction:**

No reports on atrial leadless pacing have been demonstrated in patients with the Fontan palliation. We present the case of a patient with a Lateral tunnel Fontan palliation with leadless pacing system for symptomatic bradycardia in the setting of sinus node dysfunction.

**Methods:**

After internal review board approval, a retrospective case review was performed with follow‐up of atrial leadless pacing in a patient with a Lateral tunnel Fontan.

**Results:**

A 32‐year‐old male with a medical history of tricuspid atresia status post: Blalock‐Taussig Shunt (BT) shunt, Glenn procedure, and Fontan surgery at 5 years of age presented with persistent atrial flutter and a history of symptomatic heart failure in the setting sick sinus syndrome with a dual chamber epicardial pacemaker procedure. After ablation of his intra‐atrial re‐entrant tachycardia he continued with symptomatic bradycardia in the setting of epicardial lead fracture.

Implant values demonstrated an atrial threshold of 1 Volts (V) at 0.4 ms (ms), impedance at 820 ohms and sensing at < 1 mV. He was programmed AAIR (VVIR) 80–130 bpm, rate response of 2/7, and discharged on apixaban 5 mg twice a day. Follow‐up at 4 months demonstrated no intracardiac thrombus, 98% atrial pacing, threshold of 0.75 V@0.15 ms, impedance of 590 ohms, and R‐wave of 2.5 mV. The estimated device longevity was 17.2 years.

**Conclusion:**

Atrial leadless pacing is feasible in the lateral tunnel Fontan. Larger patient population data sets are needed to assess safety of this type of pacing long‐term.

## 1 Introduction

The Fontan operation is the most utilized surgical palliation for a univentricular congenital heart disease, encompassing 0.08–0.4 per 1000 births [1, 2]. Since the 1980s surgical modifications have improved outcomes to a 30‐year survival rate of 80%–90% following a completed Fontan operation [3].

Arrhythmias including brady‐arrhythmias remain a significant aspect of morbidity in these patients, however, with rates as high as 44 percent [4]. Up to 15 percent of patients will require pacing due to this sinus node dysfunction [5]. Complications of repeat access/thoracotomies and lack of pacing site availability further complicate this problem for patients with a Fontan palliation [5]. Transvenous pacing in patients with Fontan palliation have demonstrated success in case studies only, but the high surface area of the leads remains a possible thrombus source and risk for pulmonary embolism [6, 7]. Only ventricular leadless pacing has been demonstrated in patients with the Fontan palliation including via trans‐fenestration or via carotid cutdown, however, no atrial leadless pacing has been demonstrated in patients with the Fontan palliation [8].

We report the first successful implantation of an atrial leadless pacemaker in a patient with a lateral tunnel Fontan. This was due to the patient developing symptomatic bradycardia in the setting of sinus node dysfunction.

### 1.1 Internal Review Board Review

The study was approved by the internal review board of the University of California at Davis, with consent waived, given retrospective nature of the case report.

### 1.2 Case Presentation

A 32‐year‐old male with a medical history of tricuspid atresia status post: Blalock‐Taussig Shunt (BT) shunt, Glenn procedure, and Fontan surgery at 5 years of age and with history of sick sinus syndrome with a dual chamber epicardial pacemaker procedure performed. The patient had undergone multiple pacemaker‐related procedures, including generator changes and lead revisions (3 prior epicardial sets of leads). He had an atrial lead fracture years prior to his presentation to our center.

Six years prior to presentation, the patient was diagnosed with atrial flutter with concern for tachycardia‐induced cardiomyopathy and for 2 years, he had been managed with rate control. He was hospitalized 2 years prior for decompensated heart failure with moderately reduced systemic ventricular ejection fraction (35%) and underwent IART ablation at that time. He did well and had recovery of his systemic ventricular function including an ejection fraction of 55 percent. He subsequently had lead failure of his ventricular only pacemaker. Holter monitoring did not show any significant pauses but overall heart rates lower at 48–99 bpm (average of 61 bpm) with mostly low ectopic atrial rhythm noted (Holter performed while holding metoprolol succinate for 3 days prior). Exertional dyspnea was noted with activities of daily living. Given his symptomatic bradycardia and lack of hemodynamic assessment for years, he underwent a combined cardiac catheterization and temporary atrial pacing study. Baseline hemodynamics were noted below (Table 1). With atrial pacing his hemodynamics significantly improved including Fontan pressure decreased by 17 percent, and cardiac output increase by 24 percent. Please see Table 1. Given this, the same location for temporary atrial pacing was targeted for atrial leadless pacemaker implant, which was chosen, given patient's prior failed epicardial leads and due to concern for future clot burden.

**Table 1:** Changes in Fontan, pulmonary arterial and left ventricular diastolic and systolic pressures, prior to and after pacing.

	Prior rate 55bpm	Pacing 70bpm
Fontan (mmHg)	27	20
Pulmonary artery (mmHg)	27	20
LVEDP	14	14
LVSystolic	78	98

**Figure 1:**
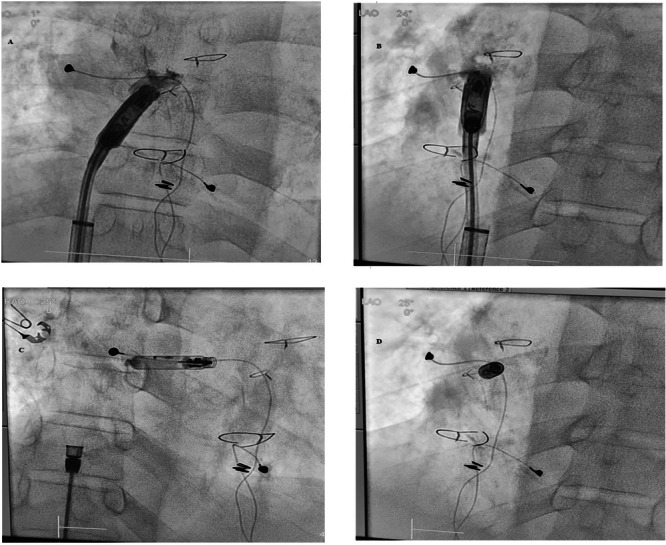
A: During implant, LAO 1 degrees, B: LAO 24 degrees, C: RAO 25 degrees, D: LAO 25 degrees.

**Figure 2:**
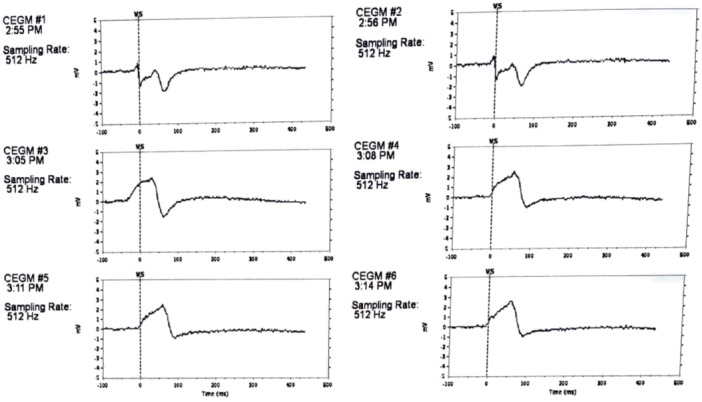
Injury pattern (EGM 1‐3: mapping, EGM 4‐6 implant injury pattern).

After ultrasound confirmation of the right femoral vein being sizable enough to accommodate the large sheath, the right femoral vein 6French (Fr) sheath (used for cardiac catheterization) was upsized serially (by 2 Fr size) to 24 Fr sheath. An Aveir delivery sheath (Abbott, Sylmar, CA, USA) was then advanced over a 0.035 Amplatz Super Stiff guidewire (Boston Scientific, Marlborough, MA, USA) into the lateral tunnel Fontan. The Aveir VR, which was chosen due to longevity, was deployed into the septal aspect of the Lateral tunnel Fontan where favorable pacing and sensing characteristics and injury pattern were noted (Figures 1 and 2). Implant values demonstrated a atrial threshold of 1 Volts (V) at 0.4 ms (ms), impedance at 820 ohms and P‐wave at 0.8 mV. He was programmed AAIR (VVIR) 80–130 bpm, rate response of 2/7, and discharged on apixaban 5 mg twice a day (planned for 6 months). Follow‐up at 7 months demonstrated no intracardiac thrombus, 98% atrial pacing, threshold of 0.75 V@0.15 ms, impedance of 600 ohms, and P‐wave of 2.6 mV. The predicted longevity at that time was noted to be 17.1 years with output at 1.25 V@0.15 ms.

## 2 Discussion

### 2.1 Atrial Leadless Pacing Is Possible in a Patient With a Lateral Tunnel Fontan

With atrial leadless pacing as well, Lateral Fontan patients may be able to have true atrioventricular synchronous dual chamber pacing. Similar results were present with transvenous atrial pacing, however, with clot burden needing consideration [7]. The Aveir AR or Aveir VR devices, with their smaller surface area compared to transvenous leads and a footprint similar to a heart failure monitoring device, may pose lower thrombotic risk requiring only short‐term anticoagulation [9].

Given the disproportionately high rate of sinus node dysfunction in the single ventricle population, and frailty of epicardial leads, other modalities of atrial pacing should be considered. Ventricular leadless pacing in Fontan patients has been demonstrated via fenestration versus carotid cut‐down, although these typically require lifelong anticoagulation [8]. Two other cases of the Aveir VR have demonstrated successful ventricular‐only pacing in Fontan patients via transbaffle puncture [10].

**Figure 3:**
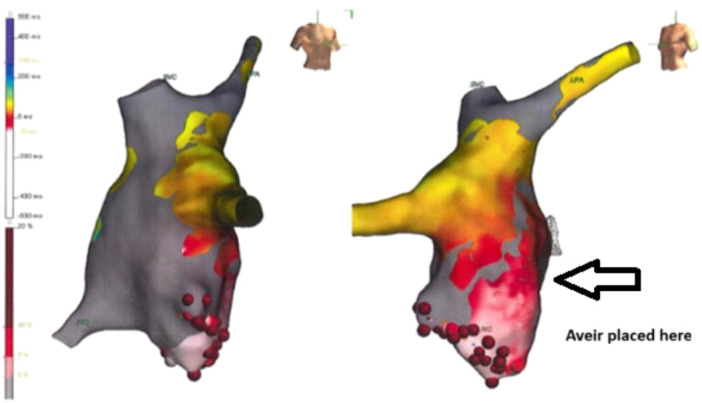
Electrophysiology study and ablation activation map of patient's intra‐atrial‐re‐entrant tachycardia (IART).

Temporary pacing during the cardiac catheterization also demonstrated lower Fontan pressures and may be an additional helpful modality when patients have borderline symptoms (in this case he was clearly symptomatic). Furthermore, in this particular patient, he had a prior electrophysiology study and ablation, and thus his favorable pacing sites were known prior, but we would recommend 3‐dimensional mapping prior to leadless pacemaker implant in patients, where pacing within baffles is performed. Figure 3 depicts his atrial flutter electrogram from the prior ablation with the area where the pacemaker was placed.

Clearly, more data is needed to support wide‐spread use of this technique, as epicardial pacing is still the most common and well‐studied pacing type in this patient population and duration of anticoagulation will need to be considered, especially in patients with open fenestrations, including possibly other types of coatings to reduce thromboses [11]. Again, thombus risk in a low‐flow environment should be considered and thus we do recommend anticoagulation for 6 months after implant.

**Figure 4:**
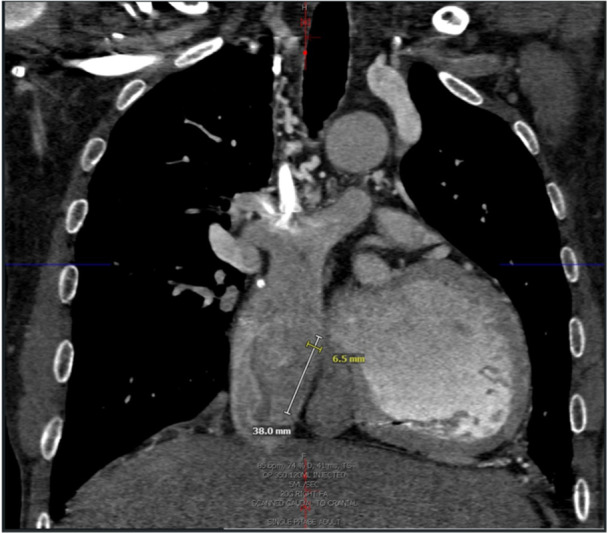
Computed tomography scan, anterior‐posterior view of the lateral tunnel Fontan with measurement length and width of the Aveir VR (38 mm by 6.5 mm).

Otherwise, one weakness of the current leadless technology is lack of electrical arrhythmia recording storage, other than the first four electrograms of atrial fibrillation mode switches only in DDD mode. There is also a lack of ventricular electrogram recording and no atrial anti‐tachycardia‐pacing is available currently as well. Furthermore, pre‐operative planning with a cardiac magnetic resonance imaging (MRI) allowed for us to consider an Aveir AR (32 mm in length) versus Aveir VR (38 mm in length), as demonstrated in Figure 4. But given the apparent reduced risk of infection in leadless devices, as well as other benefits such as reducing baffle obstruction based on smaller size, these devices may have future use in this subpopulation [12]. Additionally, echocardiographic assistance may be considered a helpful adjunct as well peri‐procedurally in these patients.

## 3 Conclusion

Stable atrial leadless pacing can be performed within the baffle of a patient's lateral tunnel Fontan without complication and with good short‐term follow‐up parameters. A larger patient population follow‐up is needed to assess safety of this type of pacing long‐term in this population.

## Conflicts of Interest

Daniel Cortez, MD, PhD, is a consultant for Abbott educational programs.

## References

[1] F. Fontan and E. Baudet , “Surgical Repair of Tricuspid Atresia,” Thorax 26, no. 3 (May 1971): 240–248, 10.1136/thx.26.3.240. 5089489 PMC1019078

[2] J. P. G. van der Ven , E. van den Bosch , A. J. C. C. Bogers , and W. A. Helbing , “State of the Art of the Fontan Strategy for Treatment of Univentricular Heart Disease,” F1000Research 7 (2018): 935, 10.12688/f1000research.13792.1. PMC6024235 30002816

[3] J. Rychik , A. M. Atz , D. S. Celermajer , et al., “Evaluation and Management of the Child and Adult With Fontan Circulation: A Scientific Statement From the American Heart Association,” Circulation 140, no. 6 Lippincott Williams and Wilkins (2019): E234–E284, 10.1161/CIR.0000000000000696. 31256636

[4] J. Rychik , A. M. Atz , D. S. Celermajer , et al., “Evaluation and Management of the Child and Adult With Fontan Circulation: A Scientific Statement From the American Heart Association,” Circulation 140 (2019): e234–e284. 31256636 10.1161/CIR.0000000000000696

[5] M. I. Cohen , N. D. Bridges , J. W. Gaynor , et al., “Modifications to the Cavopulmonary Anastomosis Do Not Eliminate Early Sinus Node Dysfunction,” Journal of Thoracic and Cardiovascular Surgery 120, no. 5 (November 2000): 891–901, 10.1067/mtc.2000.109708. 11044315

[6] W. J. Hoyt , J. P. Moore , K. M. Shannon , P. J. Kannankeril , and F. A. Fish , “Epicardial Atrial Pacing After the Extracardiac Fontan Operation: Feasibility of an Entirely Transvenous Approach,” Journal of Cardiovascular Electrophysiology 33, no. 1 (January 2022): 128–133, 10.1111/jce.15285. 34716972

[7] I. E. Assaad , T. Pastor , E. O'Leary , et al., “Atrial Pacing in Fontan Patients: The Effect of Transvenous Lead on Clot Burden,” Heart Rhythm: The Official Journal of the Heart Rhythm Society 18, no. 11 (November 2021): 1860–1867, 10.1016/j.hrthm.2021.06.1191. 34182172

[8] C. J. Goulden , D. Khanra , J. Llewellyn , A. Rao , A. Evans , and R. Ashrafi , “Novel Approaches for Leadless Pacemaker Implantation in the Extra‐Cardiac Fontan Cohort: Options to Avoid Leaded Systems or Epicardial Pacing,” Journal of Cardiovascular Electrophysiology 34, no. 11 (November 2023): 2386–2392, 10.1111/jce.16072. 37712334

[9] W. H. Marshall , S. Rajpal , M. L. Mah , et al., “Early Experience and Lessons Learned Using Implanted Hemodynamic Monitoring in Patients With Fontan Circulation,” Journal of the American Heart Association 12, no. 24 (December 2023): e031836, 10.1161/JAHA.123.031836. 38063189 PMC10863767

[10] P. Hayle , F. Altayeb , A. Hale , A. Rao , and R. Ashrafi , “Case Report Demonstrating Novel Approaches for Leadless Pacemaker Implantation in the Single Ventricle Heart,” European Heart Journal‐Case Reports 9, no. 4 (March 2025): ytaf146, 10.1093/ehjcr/ytaf146. 40302980 PMC12038896

[11] D. Hao , J. Lin , R. Liu , et al., “A Bio‐Instructive Parylene‐Based Conformal Coating Suppresses Thrombosis and Intimal Hyperplasia of Implantable Vascular Devices,” Bioactive Materials 28 (June 2023): 467–479, 10.1016/j.bioactmat.2023.06.014. 37408799 PMC10318457

[12] M. F. El‐Chami , M. Bonner , R. Holbrook , et al., “Leadless Pacemakers Reduce Risk of Device‐Related Infection: Review of the Potential Mechanisms,” Heart Rhythm: The Official Journal of the Heart Rhythm Society 17, no. 8 (August 2020): 1393–1397, 10.1016/j.hrthm.2020.03.019. 32247833

